# Comment on "A survey of poison control centers worldwide"

**DOI:** 10.1186/2008-2231-21-16

**Published:** 2013-03-07

**Authors:** Mohammad Hadi Goharbari

**Affiliations:** 1 Iran National Drug and Poison Information Center (NDPIC), Tehran, Iran; 2 Department of Toxicology and Pharmacology, Faculty of Pharmacy, Tehran University of Medical Sciences, Tehran 1417614411, Iran

## Dear Sir

The recent published article by Dr. Pourmand and his colleagues under the title of "A survey of poison control centers worldwide" was read eagerly [1]. I have had the opportunity to work for Iran National Drug and Poison Information Center (NDPIC) and also Central Staff of NDPIC for several years. I would like to state my appreciation because of consideration of Iran poison centers and also I want to comment on the Iran section of that article.

The first Iran Drug and Poison Information Center (DPIC) was founded in February of 1997 in the Food and Drug Deputy of Iran Ministry of Health and Medical Education by a group of experts and their first report of a such new activity was published by Nikfar and colleagues in the year 2000 [2]. The role of the center was to provide the best responses to questions and also consultations to healthcare professionals and public [2]. More than the enquiry service, all of the adverse reactions to medications were reported to the National Adverse Drug Reaction (ADR) center, and in order to increase the culture of rational use of drugs, the Iran DPIC participated in pharmacy and therapeutic committees; gave workshops and seminars; and produced information newsletters, pamphlets and articles [2]. At the moment, Iranian DPIC has a close collaboration with the National ADR Center and the Iranian Rational Use of Drugs (RUD) center, medical universities, and hospitals.

The Iranian DPIC developed gradually and changed its name to National Drug and Poison Information Center (NDPIC). Alongside developing of NDPIC, the other DPICs were founded in Iranian medical universities. These DPICs are mainly supported by local medical universities as well as Iran Ministry of Health and Medical Education, and currently the Food and Drug Organization. All of these centers are under the supervision of the Central Staff of NDPIC and their services are free of charge. All DPICs must report their activities and statistics to the NDPIC every three months (seasonally) [3]. In Pourmand's article, it was mentioned that there are 29 DPIC in Iran [1], while the current number of active DPICs is 34 now. According to the report of NDPIC, during the year 1390 Khorshidi (21th March 2011 – 20th March 2012) a total of 224368 telephone calls were responded by these 34 DPICs [3], a statistics seeming considerable.

There are several hospitals in different cities of Iran that have poison treatment center and serve to poisoning cases. These hospitals work under the supervision of medical universities and equipped with poisoning emergency room, intensive care unit (ICU) and specific poisoning ward [4]. The Loghman-Hakim hospital poison center that is affiliated to Shahid Behshti University of Medical Sciences is the most famous center for treatment of poisoning cases in Tehran and nearby cities [5]. This teaching equipped center is joined to a DPIC with professional experts inside the hospital [5]. It seems, Loghman-Hakim DPIC (LHDPIC) has an important role in providing specialized information for public, although, it needs more efforts to get the attention of health care professionals [5]. Of course LHDPIC is one of the 34 DPICs mentioned above. In addition to these centers, there is another specialized poison treatment center in Imam Reza hospital of Mashhad that is equipped with active DPIC and provides specialized services to the people live in Mashhad and close cities [4].

It should be noted that Iran has the potential to be a regional Center of Excellence for DPICs. To prove this claim, I would like to compare Iran DPIC with one of the well known centers in the region- Saudi Arabia DPIC. I searched literature and found an interesting report from Saudi Arabia in the region by Asiri et al [6]. In the report, authors stated that they have evaluated and analyzed the statistics of Saudi Arabia DPIC from May 2000 to December 2002, about 31 months [6]. In comparison, there is an article written by Nikfar et al. who reported activities of Iranian DPIC from February 1997 to January 2000, about 35 months [2]. Based on the Nikfar et al. article, there were 31931 enquiries which were responded and analyzed by NDPIC (about 912.3 per month) [2]. Asiri et al. reported a total of 1967 responded requests for 31 month (about 63.5 per month) [6]. There is a significant difference between the number of requests which were answered during the same period that indicates higher potential of Iranian DPIC. Of course, one of the reasons for this difference is the working time of two centers that is 24 hours a day, 365 days a year for Iranian center [3], while in Saudi Arabia NDPIC services provided from Saturday to Wednesday only in office hours [7]. Of course there are several centers in the region that can cooperate and share their data inside a network. Establishment of such a network not only would better help patients and health professional but also may provide important information for policy makers to resolve common shared matters such as misuse, abuse, or irrational use of drugs, and poisoning cases.
